# Essential Oils from the Malaysian *Citrus* (Rutaceae) Medicinal Plants

**DOI:** 10.3390/medicines3020013

**Published:** 2016-06-03

**Authors:** Siti Nur Atiqah Md Othman, Muhammad Aizam Hassan, Lutfun Nahar, Norazah Basar, Shajarahtunnur Jamil, Satyajit D. Sarker

**Affiliations:** 1 Department of Chemistry, Faculty of Science, Universiti Teknologi Malaysia, 81310 Johor Bahru, Johor, Malaysia; snamo66@yahoo.com (S.N.A.M.O.); mdzam91@gmail.com (M.A.H.); shaja@kimia.fs.utm.my (S.J.); 2 Medicinal Chemistry and Natural Products Research Group, School of Pharmacy and Biomolecular Sciences, Liverpool John Moores University, James Persons Building, Byrom Street, Liverpool L3 3AF, UK; l.nahar@ljmu.ac.uk (L.N.); s.sarker@ljmu.ac.uk (S.D.S.)

**Keywords:** *Citrus*, Rutaceae, essential oils, extraction, composition, bioactivities

## Abstract

This review article appraises the extraction methods, compositions, and bioactivities of the essential oils from the *Citrus* species (family: *Rutaceae*) endemic to Malaysia including *C. aurantifolia*, *C. grandis*, *C. hystrix*, and *C. microcarpa*. Generally, the fresh peels and leaves of the *Citrus* species were extracted using different methods such as steam and water distillation, Likens-Nikerson extraction, solvent extraction, and headspace solid-phase micro-extraction (HS-SPME). Most of the *Citrus* oils were found to be rich in monoterpene hydrocarbons with limonene (**1**) as the major component identified in the peels of *C. aurantifolia* (39.3%), *C. grandis* (81.6%–96.9%), and *C. microcarpa* (94.0%), while sabinene (**19**) was the major component in the peels of *C. hystrix* (36.4%–48.5%). In addition, citronellal (**20**) (61.7%–72.5%), linalool (**18**) (56.5%), and hedycaryol (**23**) (19.0%) were identified as the major components in the oil of *C. hystrix* leaves, *C. grandis* blossom and *C. microcarpa* leaves, respectively. The *C. hystrix* essential oil has been experimentally shown to have antimicrobial and antifeedant activities, while no bioactivity study has been reported on the essential oils of other Malaysian *Citrus* species.

## 1. Introduction

The *Citrus* species of the family *Rutaceae* provide several popular edible fruits in the world. The species are widely distributed in the Indo-Malaysia region, South-East Asia, India and China, but cultivated worldwide [1]. The fruits and the leaves of the *Citrus* species contain a variety of essential oils with various distinct flavors, and biologically-active compounds, which are important to human nutrition and diet, which include vitamin C, folic acid, potassium, flavonoids, coumarins, pectin, and dietary fibers. In Malaysia, the oils from the fruits and the leaves are commercially used as flavors and fragrances, as well as in cooking, perfumery and medical treatments, especially in aromatherapy [2]. Recent studies on the Malaysian *Citrus* plants have reported the identification and composition of essential oils of several *Citrus* species including *C. aurantifolia*, *C. grandis*, *C. hystrix*, and *C. microcarpa*. A monoterpene hydrocarbon, limonene (**1**), is the major component in the essential oils from the peels of these Malaysian *Citrus* species [3,4,5]. This review focuses on the details of extraction methods, identification and composition of essential oils from the Malaysian *Citrus* species and also on their biological properties.

## 2. Essential Oils from the Malaysian *Citrus* Species

### 2.1. Citrus aurantifolia (Cristm.) Swingle

**Figure 1:**
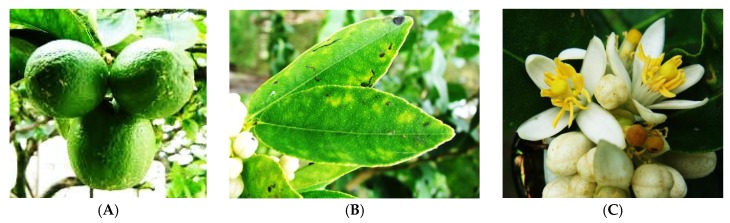
The (**A**) fruit; (**B**) leaf; and (**C**) flower of *C. aurantifolia*. (Photos credit: Forest Starr and Kim Starr).

*Citrus aurantifolia* (Figure 1), commonly known as ‘limau nipis’ or ‘common lime’ is the most popular *Citrus* species in Malaysia. Usually, *C. aurantifolia* is used in cuisine and traditional medicine. It is a spiny stem plant which is about 3–5 m tall. This plant has ovate-shaped 5–9 cm long leaves with 3–5 cm thickness. The flowers of *C. aurantifolia* are white and the fruits are green and turn yellow after ripe with a diameter of 3–6 cm [6]. The essential oil from *C. aurantifolia* contains a variety of monoterpene and sesquiterpene hydrocarbons, and limonene (**1**) is the most abundant one. Traditionally, *C. aurantifolia* is used to aid digestion process, and to reduce sugar, fat, and cholesterol in blood [7]. The oil extracted from the fruits can be used for cold, asthma, arthritis, and bronchitis [8]. The fruit juice is used as a facial wash to refresh the skin and prevent pimples, increase stamina, treat dysfunctional uterine bleeding, and act as an antidote for poison [9,10]. The juice also has been found to be an excellent cough reliever when added with sugar and honey. Moreover, it can also reduce body temperature, remove body smell and act as a softener for meat [6]. Additionally, it also has been useful as mosquito, cat, and moth repellants [11,12]. *C. aurantifolia* has been reported to have biological activities, e.g., antioxidant and anti-inflammatory [7].

**Figure 2:**
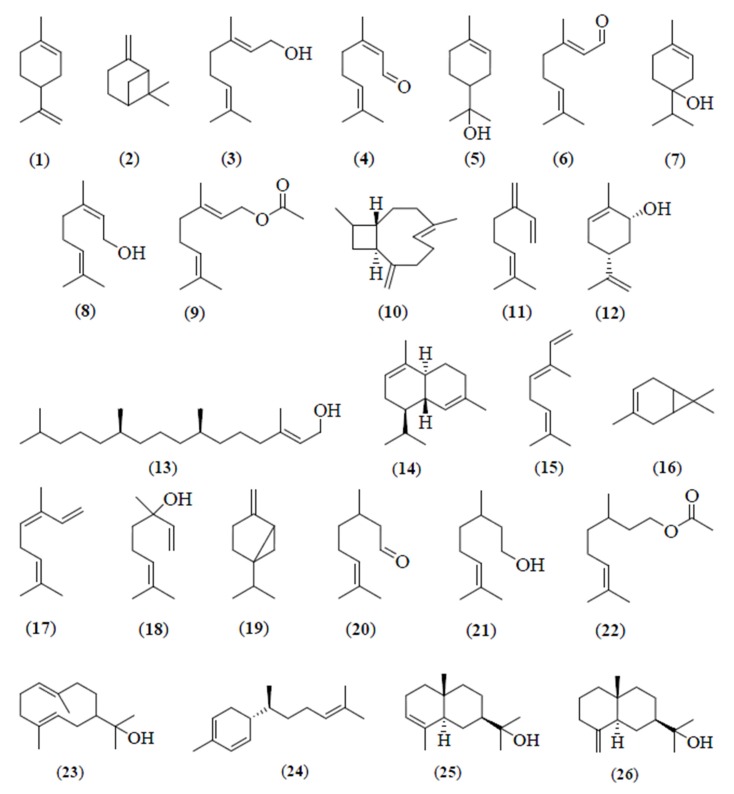
Chemical structures of the components identified from the *Citrus* essential oils.

The essential oil of *C. aurantifolia* peels from Masjid Tanah, Melaka, was reported to contain limonene (**1**) (39.3%), β-pinene (**2**) (28.4%), geraniol (**3**) (7.5%), neral (**4**) (5.3%), α-terpineol (**5**) (2.4%), geranial (**6**) (2.1%), and terpinen-4-ol (**7**) (2.0%) (Table 1) [3]. Geranial (**6**) (19.4%), limonene (**1**) (16.4%), neral (**4**) (11.4), nerol (**8**) (9.5%), geraniol (**3**) (7.5%), geranyl acetate (**9**) (6.6%), and β-caryophyllene (**10**) (5.7%) (Figure 2) were the major compounds in the essential oil of the leaves of *C. aurantifolia* [3].

### 2.2. Citrus grandis L. Osbek

**Figure 3:**
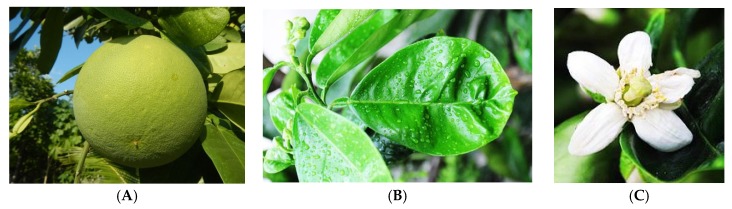
The (**A**) fruit; (**B**) leaf; and (**C**) flower of *C. grandis*. (Photos kredit: Judgefloro, Davidals, Amada44).

*Citrus grandis* L. (synonyms: *C. decumana* L. and *C. maxima* Merr.) (Figure 3) is one of the most popular fruits in Malaysia, especially in Tambun, Perak. It is native to Malaysia, and also other countries including Bangladesh, India, Indonesia, Philippines, Thailand, and Vietnam, and grows widely in Malaysia on the tailings of tin mines. In Malaysia, *C. grandis* has a variety of local names, such as ‘limau bali’, ‘limau abong’, ‘limau besar’, ‘limau betawi’, ‘limau bol’ and ‘limau jambua’. This plant is also known as ‘pumelo’ or ‘pummelo’, with a height of 5–15 m, and the thickness of this plant is 10–30 cm. The leaves of pumelo are dotted, glandular, alternate, ovate and elliptic, 5–20 cm long and 2–12 cm thick. The fruits of pumelo are pear-shaped with a width of 10–30 cm and pale-yellow or greenish yellow color [13,14]. *C. grandis* is well known for its therapeutic values; it can cure fever, gout, arthritis, kidney disorders and ulcers [15]. The fruit pulp and peels are used as an appetizer, stomach-tonic, and also for the treatment of inflammation and cough. The fruit juice has potential in influencing weight loss and promoting cholesterol reduction. In addition, *C. grandis* fruits are also used in the food, cosmetic, perfume and pharmaceutical industries as a flavoring or fragrance-enhancing agent [16]. The essential oil from the fruits and the leaves of *C. grandis* is used as one of the components of various toiletry products. Highly aromatic character of its flowers is routinely exploited by perfume manufactures [14].

Steam distillation method yielded limonene (**1**) (81.6%) as the most abundant constituent in the pumelo peel essential oil, while β-myrcene (**11**) (2.2%), and *cis*-carveol (**12**) (1.5%) (Figure 2) were reported as the minor components [4]. However, the Likens-Nikerson method provided slightly higher yield of limonene (**1**) (86.8%), followed by β-myrcene (**11**) (1.6%) and *cis*-carveol (**12**) (1.4%) [4]. Comparison of the constituents of pumelo peel oil from Kepong, Selangor, was carried out and limonene (**1**) (95.1%) was reported as the major and β-myrcene (**11**) (1.6%) as the minor components. Sesquiterpene hydrocarbons were identified as the principal components in the leaf essential oil of *C. grandis* including phytol (**13**) (23.1%), β-caryophyllene (**10**) (15.4%) and α-cadinene (**14**) (7.1%). Minor monoterpene hydrocarbons were *trans*-β-ocimene (**15**) (9.9%), β-pinene (**2**) (4.9%), geranial (**6**) (4.5%), and δ-3-carene (**16**) (3.9%) (Figure 2) (Table 1) [3]. The essential oil of the fruits peels from the white and the pink pomelo is a major source of limonene (**1**) (93%–97%). Two methods of extraction were employed for the extraction of essential oil from the white and the pink pomelo peels; head-space solid-phase micro-extraction (HS-SPME) and solvent extraction. The essential oil of the blossoms was extracted by the HS-SPME method. The essential oil of the blossoms of white pomelo was found to be rich in limonene (**1**) (48.2%), and also contained *cis*-β-ocimene (**17**) (12.0%), and linalool (**18**) (9.2%) (Figure 2). However, the pink pomelo oil was reported to contain an abundant of linalool (**18**) (56.5%), and also limonene (**1**) (15.5%) and *cis*-β-ocimene (**17**) (4.0%) [5].

### 2.3. Citrus hystrix

**Figure 4:**
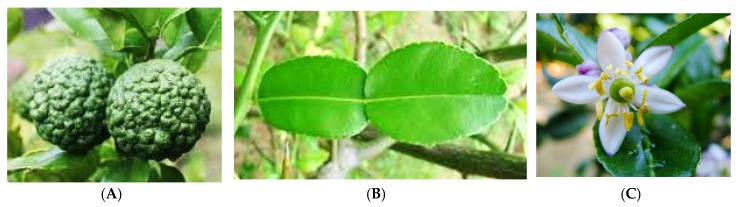
The (**A**) fruit; (**B**) leaf; and (**C**) flower of *C. hystrix*. (Photos kredit: Robyn Jay, Forest Starr and Kim Starr, David Rofas).

*Citrus hystrix* (synonym: kaffir lime) (Figure 4) is known as ‘limau purut’ or ‘wild lime’. *C. hystrix* leaves and fruits are widely used as spices in preparation of ‘tomyam’, either white or red, and it is famous dish in Malaysia and Thailand [22]. The height of this plant is about 3–5 m and the fragrant green leaves are 7.5–10 cm long. It has white flowers with 4–6 petals. The diameter of pear-shaped fruits is about 5.0–7.5 cm with wrinkle on the surface of fruit. The fruit is dark green, and yellow when ripe [23]. The essential oil of *C. hystrix* is used in aromatherapy and an essential ingredient of various cosmetic and beauty products [20].

In traditional medicine, *C. hystrix* is used to treat flu, fever, hypertension, abdominal pains, and diarrhea in infants [24]. The fruits are used as a digestive stimulant, blood purifier, and reduce high blood pressure [25,26]. Additionally, the fruits are used in cooking for flavoring and also in the production of shampoo as an insecticide for washing the head [27]. In addition, the fruit juice is used in softening the skin and the mixture of the fruit juice with bath water can be used to eliminate body odor [28]. Furthermore, the essential oil of *C. hystrix* has been reported to have various bioactivities such as antioxidant, antibacterial, antileukimic, and antitussive [26].

The essential oil of kaffir lime peel from Dengkil, Selangor, contained sabinene (**19**) (36.0%–49.0%), limonene (**1**) (17.0%–33.0%), citronellal (**20**) (3.0%–11.0%) (Figure 2) and β-pinene (**2**) (8.0%–14.0%) as major components. Three methods were used to extract kaffir lime peel essential oil, e.g., hydro-diffusion steam distillation system, steam distillation with induction heating system, and automated steam distillation process with optimized temperature at 90 °C [17,18,19]. However, citronellal (**20**) (66.9%) and β-citronellol (**21**) (6.6%) (Figure 2) were the major components in kaffir lime peel oil from Selangor, obtained using the hydro-distillation method [29].

The essential oil of *C. hystrix* fresh leaves from Jerangau, Terengganu, extracted by the steam distillation and the Likens-Nikerson extraction methods, was found to be dominated by citronellal (**20**) (61.0%–73.0%), β-citronellol (**21**) (10.0%–14.0%), and limonene (**1**) (5.0%–7.0%) as major components (Table 1). β-Pinene (**2**) (23.5%) and sabinene (**19**) (20.1%) appeared as the major components of *C. hystrix* peel, followed by citronellal (**20**) (12.6%), limonene (**1**) (11.8%), and β-citronellol (**21**) (3.3%) [21]. Moreover, β-pinene (**2**) (39.3%), limonene (**1**) (14.2%), citronellal (**20**) (11.7%), and terpinen-4-ol (**7**) (8.9%) were identified as the principal components in kaffir lime peels from Masjid Tanah, Melaka. However, citronellal (**20**) (72.4%), β-citronellol (**21**) (6.7%), and citronellyl acetate (**22**) (4.1%) (Figure 2) were reported to be the major components in kaffir lime leaves, followed by β-pinene (**2**) (1.9%) and limonene (**1**) (0.1%) as minor components. Water distillation was used as a method to extract kafir lime peels and leaves from Masjid Tanah, Melaka [3].

The antibacterial susceptibility of the essential oils and oil emulsions of Malaysian *C. hystrix* was evaluated against *Escherichia coli*, *Bacillus subtilis*, and *Staphylococcus aureus* using the disc diffusion method. Pure essential oil with a percentage of 2% by weight exhibited a strong inhibitory effect against *E. coli* and *B. subtilis* with the zones of inhibition of 16.0 and 15.0 mm, respectively. Meanwhile, the formulated emulsions with surfactant mixture of Tween 80 and Span 80 (90:10) with 2% (by weight) essential oil displayed the most potential antibacterial activity against *E. coli* with the zones of inhibition ranging between 11.0 to 18.0 mm [2].

The topical application bioassay on uniform weighted second instar larvae in the laboratory was carried out to determine the insecticidal properties of the essential oil from the leaves of the Malaysian *C. hystrix* against *Spodoptera litura* (tobacco army worm). The study demonstrated considerable repellant activity of *C. hystrix* essential oil against the *S. litura* larvae after 24 and 48 h of treatment with LD_50_ values of 29.25 and 26.75 μg/mL, respectively [29].

### 2.4. Citrus microcarpa (Bunge) Wijnands

**Figure 5:**
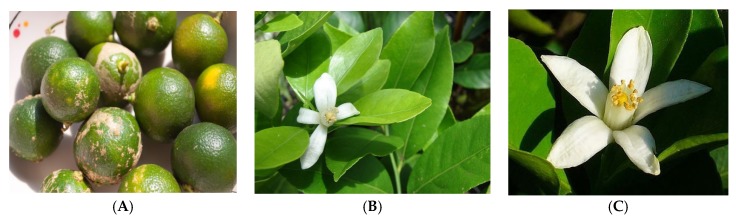
The (**A**) fruit; (**B**) leaf and (**C**) flower of *C. microcarpa*. (Photos kredit: Ronald Escanlar, Forest Starr and Kim Starr, H. Zell).

*Citrus microcarpa* (synonym: *C. madurensis*) (Figure 5), common name: ‘limau kasturi’ in Malaysia, is used in the preparation of beverages. *C. microcarpa* is 3–5 m tall with abundant of long spine on the stem, branches and twigs. The dark green leaves of *C. microcarpa* are between 2.5–6.8 cm long and 2–3 cm thick. The round or oblong-shaped green leaves of this plant are 2.5–3.8 cm in diameter. This plant is used to treat fever, cough, and pharyngitis [30]. The juice is traditionally used to prevent respiratory diseases, strengthen the bones and act as growth stimulant for children. The juice is also commonly used in cooking as flavoring ingredients and additives. The leaves of this plant can be used in the treatment of skin diseases, relieve headache and also act as a mouth wash to treat sore throat [31]. Essential oil from *C. microcarpa* is used commercially in perfumes, food, cosmetics and detergents. It is one of the ingredients in pharmaceutical, aromatherapy and antiseptic products [32].

The essential oil from *C. microcarpa* peels was reported to be rich in limonene (**1**) (94.0%) similar to *C. aurantifolia*. β-Myrcene (**11**) (1.8%), linalool (**18**) (0.4%), and α-terpineol (**5**) (0.3%) were detected as the minor components (Table 1) in the oil. Sesquiterpene hydrocarbons were the most abundant in the leaves of *C. microcarpa*. These include hedycaryol (**23**) (19.0%), α-sesquiphellandrene (**24**) (18.3%), α-eudesmol (**25**) (14.4%) and β-eudesmol (**26**) (8.6%) (Figure 2). The essential oil was extracted by hydrodistillation for 8 h similar to that of *C. grandis* and *C. aurantifolia* oils [3].

## 3. Conclusions

Extraction and identification of the essential oils from the Malaysian *Citrus* species showed that limonene (**1**) (96.9%) and sabinene (**19**) (48.5%) were the major components in *C. grandis* and *C. hystrix*, respectively. Sample collections from different locations, and differences in extraction methods resulted in different composition and percentage of yields. Moreover, extraction of essential oils from different parts of *Citrus* plants also gave different major components. The bioactivity studies on the *C. hystrix* essential oil revealed strong antimicrobial activity against *E. coli* and good antifeedant properties against *S. litura*. More bioactivity studies on the essential oils of the Malaysian *Citrus* plants need to be carried out to acquire better bioactivity profiles of these oils.

## Figures and Tables

**Table 1:** Composition of Essential Oil of *Citrus* Species in Malaysia.

Plant	Location	Method	Part	Components								Ref.
				Monoterpene Hydrocarbon	%	Oxygenated Monoterpene	%	Sesquiterpene Hydrocarbon	%	Oxygenated Sesquiterpene	%	
*Citrus aurantifolia* (Cristm.) Swingle	Masjid Tanah, Melaka	Water Distillation	Peel	α-Pinene	1.5	Terpinen-4-ol	2.0	β-Caryophyllene	0.8	(*Z*)-Nerolidol	0.6	[3]
				β-Pinene	28.4	α-Terpineol	2.4	α-Bergamotene	0.4	α-Eudesmol	0.1	
				β-Myrcene	1.0	Neral	5.3	α-Humulene	0.1	β-Eudesmol	0.1	
				δ-3-Carene	0.5	Geraniol	7.5	(*Z*)-β-Farnesene	0.4	Elemol	0.1	
				Limonene	39.3	Geranial	2.1	(*E*)-β-Farnesene	1.5			
				γ-Terpinene	0.8	Geranyl acetate	0.6					
			Leaves	Sabinene	0.1	Linalool	1.1	(*Z*)-β-Farnesene	0.1	Phytol	1.0	
				β-Pinene	0.9	Nerol	9.5	α-Humulene	0.8	(*Z*)-Nerolidol	2.1	
				β-Myrcene	0.8	Neral	11.4	α-Guaiene	0.9	α-Eudesmol	0.3	
				Limonene	16.4	Geraniol	7.5	β-Caryophyllene	5.7	β-Eudesmol	0.3	
				*cis*-β-Ocimene	0.4	Geranial	19.4	(*E*)-β-Farnesene	1.8	Elemol	0.5	
				*trans*-β-Ocimene	1.9	Geranyl acetate	6.6	β-Bisabolene	0.1			
*Citrus grandis* L. Osbek	Tambun, Perak	Steam Distillation	Peel	α-Pinene	0.3	Linalool	0.8	δ-Guaiene	tr	Caryophyllene oxide	tr	[4]
				Sabinene	0.1	Terpinen-4-ol	0.1	α-Cubebene	0.1	Muurolol	0.1	
				β-Pinene	0.1	*cis*-Carveol	1.5	*trans*-Caryophyllene	tr	Farnesol	0.3	
				β-Myrcene	2.2	*trans*-Carveol	0.6	Aromadendrene	tr	Nootkatone	0.3	
				Limonene	81.6	1-α-Terpineol	1.2	δ-Cadinene	tr			
				β-Cymene	0.1	Carvone	0.9					
		Likens-Nikerson Extraction	Peel	α-Pinene	0.1	Linalool	0.5	δ-Guaiene	tr	Caryophyllene oxide	tr	
				Sabinene	0.1	Terpinen-4-ol	0.3	α-Cubebene	tr	Muurolol	tr	
				β-Pinene	0.1	*cis*-Carveol	1.4	*trans*-Caryophyllene	0.1	Farnesol	0.3	
				β-Myrcene	1.6	*trans*-Carveol	0.4	Aromadendrene	tr	Nootkatone	0.3	
				Limonene	86.8	1-α-Terpineol	1.1	δ-Cadinene	0.1			
				β-Cymene	tr	Carvone	0.7					
	Kepong, Selangor	Water Distillation	Peel	β-Pinene	0.6	Linalool	0.2	(*Z*)-β-Farnesene	0.3	α-Eudesmol	tr	[3]
				α-Pinene	0.3	Terpinen-4-ol	0.1	α-Guaiene	0.1	Phytol	0.1	
				β-Myrcene	1.6	α-Terpineol	0.2	(*E*)-β-Farnesene	0.1			
				α-Phellandrene	0.1	Neral	0.1	Aromadendrene	tr			
				Limonene	95.1	Geraniol	0.1	β-Caryophyllene	0.1			
				γ-Terpinene	0.1	Geranial	0.1					
			Leaves	Sabinene	0.1	Terpinolene	1.6	β-Caryophyllene	15.4	Hedycaryol	2.4	
				β-Pinene	4.9	Nerol	1.5	α-Humulene	1.8	(*Z*)-Nerolidol	0.8	
				δ-3-Carene	3.9	Neral	4.5	(*Z*)-β-Farnesene	2.2	α-Eudesmol	1.8	
				Limonene	1.4	Geraniol	1.4	α-Cadinene	7.1	β-Eudesmol	1.6	
				*cis*-β-Ocimene	0.8	Geranial	4.5	γ-Cadinene	0.9	Phytol	23.1	
				*trans*-β-Ocimene	9.9	Citronellyl acetate	1.8					
*Citrus grandis* L. Osbek (white pomelo)	Tambun, Perak	HS-SPME	Blossom	β-Pinene	0.3	Linalool	9.2	β-Caryophyllene	0.1	*trans*-Nerolidol	0.1	[5]
				δ-3-Carene	0.6	α-Terpineol	tr	α-Humulene	0.1	*cis*-Farnesol	0.4	
				α-Terpinene	2.8	Citronellol	0.3	β-Farnesene	0.1	Spathulenol	tr	
				Limonene	48.2	Nerol	1.5	Germacrene D	0.1			
				*cis*-β-Ocimene	12.0	Geraniol	1.3	β-Bisabolene	0.1			
				allo-Ocimene	1.6	Carveol	0.1	α-Farnesene	0.1			
		HS-SPME	Peel	β-Pinene	0.1	Linalool	0.2	β-Caryophyllene	0.1	*trans*-Nerolidol	tr	
				δ-3-Carene	0.1	α-Terpineol	tr	α-Humulene	tr	*cis*-Farnesol	tr	
				α-Terpinene	tr	Citronellol	tr	β-Farnesene	0.1	Elemol	0.1	
				Limonene	96.9	Nerol	0.1	Germacrene D	tr			
				*cis*-β-Ocimene	0.1	Geraniol	0.1	β-Bisabolene	tr			
				β-Myrcene	0.2	Carveol	tr	α-Farnesene	0.1			
		Solvent extraction	Peel	β-Pinene	0.1	Linalool	0.2	β-Caryophyllene	0.1	*trans*-Nerolidol	0.1	
				δ-3-Carene	0.1	α-Terpineol	0.1	α-Humulene	0.1	*cis*-Farnesol	0.1	
				α-Terpinene	0.1	Citronellol	tr	β-Farnesene	0.1	Elemol	0.1	
				Limonene	95.4	Nerol	0.2	Germacrene D	0.1			
				*cis*-β-Ocimene	0.1	Geraniol	0.3	β-Bisabolene	0.1			
				β-Myrcene	0.1	Carveol	0.1	α-Farnesene	0.1			
*Citrus grandis* L. Osbek (pink pomelo)	Tambun, Perak	Headspace solid phase microextraction (HS-SPME)	Blossom	β-pinene	0.1	Linalool	56.5	β-Caryophyllene	0.1	*trans*-Nerolidol	0.1	[5]
				δ-3-carene	0.2	α-Terpineol	tr	α-Humulene	0.1	*cis*-Farnesol	1.8	
				α-Terpinene	2.5	Citronellol	0.2	β-Farnesene	0.1	Spathulenol	tr	
				Limonene	15.5	Nerol	0.4	Germacrene D	0.1			
				*cis*-β-Ocimene	4.0	Geraniol	0.4	β-Bisabolene	0.1			
				allo-Ocimene	1.1	Carveol	0.1	α-Farnesene	0.2			
		HS-SPME	Peel	β-Pinene	0.1	Linalool	0.1	β-Caryophyllene	0.1	*trans*-Nerolidol	0.1	
				δ-3-Carene	0.1	α-Terpineol	0.1	α-Humulene	tr	*cis*-Farnesol	tr	
				α-Terpinene	tr	Citronellol	0.1	β-Farnesene	0.1	Elemol	0.1	
				Limonene	96.1	Nerol	0.1	Germacrene D	tr			
				*cis*-β-Ocimene	0.1	Geraniol	0.1	β-Bisabolene	tr			
				β-Myrcene	0.4	Carveol	tr	α-Farnesene	0.1			
		Solvent extraction	Peel	β-Pinene	0.1	Linalool	0.3	β-Caryophyllene	0.2	*trans*-Nerolidol	0.1	
				δ-3-Carene	tr	α-Terpineol	0.2	α-Humulene	0.1	*cis*-Farnesol	0.1	
				α-Terpinene	0.1	Citronellol	0.1	β-Farnesene	0.1	Elemol	0.1	
				Limonene	93.1	Nerol	0.2	Germacrene D	0.2			
				*cis*-β-Ocimene	0.1	Geraniol	0.3	β-Bisabolene	tr			
				β-Myrcene	0.1	Carveol	0.1	α-Farnesene	0.1			
*Citrus hystrix* D.C.	Dengkil, Selangor	Hydro-difusion steam distillation system	Peel	α-Thujene	0.2	Linalool	0.8	α-Copaene	0.5			[17]
				α-Pinene	1.8	Citronellal	10.8	Caryophyllene	0.3			
				Sabinene	36.4	Terpinen-4-ol	1.1	α-Humulene	0.1			
				β-Pinene	8.6	α-Terpineol	0.6	Germacrene D	0.4			
				β-Myrcene	1.7	Citronellol	1.8	β-Selinene	0.1			
				Limonene	32.5			δ-Cadinene	0.4			
		Steam distillation with induction heating system	Peel	α-Thujene	0.1	Linalool	0.1	α-Copaene	0.1			[18]
				α-Pinene	3.2	Citronellal	3.3	Germacrene D	0.2			
				Sabinene	48.5	Terpinen-4-ol	0.5	δ-Cadinene	0.1			
				β-Pinene	10.1	α-Terpineol	0.2					
				β-Myrcene	1.5	Citronellyl acetate	0.1					
				Limonene	27.7							
		Automated steam distillation process	Peel	α-thujene	0.2	Linalool	1.2					[19]
				α-pinene	3.3	Citronellal	7.8					
				Sabinene	46.6	Terpinen-4-ol	2.4					
				β-pinene	13.5	α-Terpineol	0.9					
				β-Myrcene	1.8							
				Limonene	17.2							
	Selangor	Hydro-distillation	Leaves	Sabinene	0.2	Linalool	3.9			Nerolidol	0.1	[20]
				β-Myrcene	0.1	Citronellal	66.9					
				(*E*)-2,5-Dimethyl-1,6-octadine	0.1	Isopregol	0.7					
				*cis*-2,6-Dimethyl-2,6-octadine	0.3	β-Citronellol	6.6					
						Citronellol	1.8					
						Geraniol	0.4					
	Jerangau, Terengganu	Steam Distillation	Fresh Leaves	α-Pinene	0.1	Linalool	1.0	*trans*-Caryophyllene	tr	Elemol	tr	[21]
				Sabinene	1.6	Citronellal	61.7	β-Elemene	tr	Nerolidol	1.2	
				β-Pinene	0.1	β-Citronellol	13.4	α-Muurolene	tr	Guaiol	0.2	
				β-Myrcene	0.7	*iso*-Pulegol	0.9	β-Bisabolene	tr	Caryophyllene oxide	tr	
				Limonene	5.9	Citronellyl acetate	2.0	δ-Cadinene	tr			
				*p*-Cymene	0.1							
		Likens-Nikerson Extraction	Fresh Leaves	α-Pinene	0.1	Linalool	1.6	δ-Cadinene	tr	Elemol	tr	[21]
				Sabinene	2.0	Citronellal	72.5			Nerolidol	tr	
				β-Pinene	0.1	β-Citronellol	10.3			Guaiol	tr	
				β-Myrcene	0.6	*iso*-Pulegol	1.2					
				Limonene	6.8	Citronellyl acetate	1.2					
				*p*-Cymene	tr							
		Likens-Nikerson Extraction	Peel	α-Pinene	1.7	Linalool	1.8	β-Bisabolene	1.2	Elemol	tr	[21]
				Sabinene	20.0	Citronellal	12.6	δ-Cadinene	0.6	Nerolidol	0.2	
				β-Pinene	23.5	β-Citronellol	3.3			Guaiol	0.1	
				β-Myrcene	1.0	*iso*-pulegol	0.5			Caryophyllene oxide	0.1	
				Limonene	11.8	Citronellyl acetate	1.7					
				*p*-cymene	0.3							
	Masjid Tanah, Melaka	Water Distillation	Peel	α-Pinene	2.0	*cis*-Linalool oxide	1.9	β-caryophyllene	0.4	Hedycaryol	0.3	[3]
				β-Pinene	39.3	Terpinolene	1.6	α-Humulene	0.1	(*Z*)-Nerolidol	0.1	
				Limonene	14.2	Linalool	1.9	(*Z*)-β-Farnesene	0.2	α-Eudesmol	0.2	
				β-Myrcene	1.3	Terpinen-4-ol	8.9	α-Cadinene	0.1	β-Eudesmol	0.2	
				δ-3-Carene	1.4	Citronellal	11.7	(*E*)-β-Farnesene	0.1	Phytol	0.1	
				γ-Terpinene	2.4			δ-Cadinene	0.5	α-Sinensal	0.1	
			Leaves	β-Pinene	1.9	*trans*-Sabinene hydrate	1.5	β-Cubebene	0.2	Hedycaryol	0.3	[3]
				β-Myrcene	0.9	Linalool	1.7	β-Caryophyllene	0.9	(*Z*)-Nerolidol	0.9	
				δ-3-Carene	0.1	Citronellal	72.4	α-Cadinene	0.4	α-Eudesmol	0.2	
				Limonene	0.1	Citronellol	6.7	α-Humulene	0.2	β-Eudesmol	0.2	
				*trans*-β-Ocimene	0.5	Citronellyl acetate	4.1	(*E*)-β-Farnesene	0.2	Elemol	0.3	
				γ-Terpinene	0.3	Geranyl acetate	0.8	δ-Cadinene	0.4			
*Citrus microcarpa* (Bunge) Wijnands	Masjid Tanah, Melaka	Water Distillation	Peel	α-Pinene	0.5	δ-Elemene	0.1	β-Caryophyllene	tr	Elemol	0.1	[3]
				β-Pinene	0.1	Linalool	0.4	(*Z*)-β-Farnesene	0.7	β-Eudesmol	0.2	
				Myrcene	1.8	Terpinen-4-ol	0.1	Aromadendrene	0.1			
				α-Phellandrene	0.1	α-Terpineol	0.3	(*E*)-β-Farnesene	0.1			
				Limonene	94.0	Terpinolene	0.1	α-Guaiene	0.1			
				γ-Terpinene	0.1	Geranyl acetate	0.2					
			Leaves	α-Pinene	0.8	δ-Elemene	2.7	β-Caryophyllene	2.8	Hedycaryol	19.0	[3]
				β-Pinene	13.4	Linalool	6.1	α-Humulene	0.6	(*Z*)-Nerolidol	1.2	
				Myrcene	0.2	Terpinen-4-ol	0.4	α-Sesqui-phellandrene	18.3	α-Eudesmol	14.4	
				α-Phellandrene	0.8	α-Terpineol	0.3	α-Selinene	1.8	β-Eudesmol	8.6	
				Limonene	0.7	β-Elemene	1.1	δ-Cadinene	0.5	Elemol	0.6	
				*trans*-β-Ocimene	2.0	Geranyl acetate	0.1			Phytol	0.4	

tr: trace level.
